# Pathology Seen in Myenteric Plexus in Two Subjects With Waardenburg Syndrome

**DOI:** 10.1111/nmo.70073

**Published:** 2025-05-13

**Authors:** Björn Ersson, Elisabet Gustafson, Johan Danielson, Irina Alafuzoff

**Affiliations:** ^1^ Department of Pathology Uppsala University Hospital Uppsala Sweden; ^2^ Department of Pediatric Surgery Uppsala University Hospital Uppsala Sweden; ^3^ Genetics and Pathology, Institution of Immunology Uppsala University Uppsala Sweden

**Keywords:** immunohistochemistry (IHC), pediatric intestinal pseudo‐obstruction (PIPO), SOX10, Waardenburg syndrome

## Abstract

**Objectives:**

The aim was to assess the neuroglial compartment in the myenteric plexus of two subjects with genetically verified Waardenburg syndrome (WS) type 4 (WS4) and to compare the outcome with four “age‐matched” controls.

**Design:**

Gut samples from four control cases and from two newborn subjects with WS4, one with peripheral demyelinating neuropathy, dysmyelinating leukodystrophy, WS and Hirschprung disease (PCWH) (*SOX10*, c.769A>T, p.Lys257*) and one with Waardenburg‐Shah syndrome (WSS) (*EDN3*, c.472C>T,p.Arg158Cys)—were assessed histologically and immunohistochemically. Antibodies directed to glial cells (SOX10), ganglion cells (HuC/D), and interstitial cells of Cajal (CD117) were applied.

**Results:**

For the child with PCWH syndrome, both the small and large intestine showed a reduction in the number of glial cells (SOX10), in parallel with hypoganglionosis (HuC/D), when compared with “age‐matched” controls. In the child with WSS, a severe reduction in the number of glial cells (SOX10) was observed in both the small and large intestine accompanied by aganglionosis (HuC/D) with a skipped segment. The number of interstitial cells of Cajal (CD117) appeared unaffected in both PCWH and WSS cases.

**Conclusion:**

A severe reduction of glial cells and a severe reduction or loss of ganglion cells (the number of cells assessed per unit length), were seen in our study subjects when compared with “age‐matched” controls. Contrary to the above the presence of Cajal cells was unaffected.

## Graphical abstract

Ganglion cells immunohistochemically labeled with pan‐neuronal marker HuC/D in sections from ileum. Note the different number of stained cells in the myenteric plexus. A, control subject, B, patient with *SOX10* mutation (hypoganglionosis) C, patient with *EDN3* mutation (aganlionosis). Both patients diagnosed with Waardenburg syndrome type 4 including gut dysmotility.

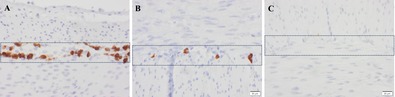

**Summary**

- Two mutations, one of *SOX10* and one of *EDN3*, cause Waardenburg Syndrome type 4.
- These mutations lead to different phenotypes.
- Histopathological assessment of the neuroglial compartment in the myenteric plexus in the gut of these two patients reveals different alterations which likely cause their clinically observed motility disorder.
- Tissue samples from patients with gastrointestinal motility disorders are warranted to further our understanding of these complex diseases.

## 1 Introduction

Gastrointestinal (GI) motility disorders in children are relatively common, with the vast majority being functional [1, 2]. In 5% of children, the GI motility disorder is presumed to have an underlying organic cause [1, 2, 3]. Histologically the underlying cause has been shown to be related to visceral myopathy and/or visceral neuropathy, with neuropathic disorders being more common [1, 3].

One histologically defined type of GI motility disorder primarily affecting the large bowel is Hirschsprung disease (HSCR), a developmental disorder characterized by segmental absence of ganglion cells, aganglionosis, in the submucosal and myenteric plexus (MP) [4]. Parallel with aganglionosis, hypertrophic nerves can be seen in the rectum [4, 5]. The aganglionic segment starts distally and extends proximally for various lengths [6]. Very few of HSCR cases display aganglionosis in the proximal small intestine [4, 6]. Noteworthy, a subtype of HSCR with total colonic aganglionosis (TCA) and near total intestinal aganglionosis has been described to affect about 10% of all patients with HSCR [6, 7]. In addition, an unusual entity of “skip segment” HSCR has been described where a segment of normally ganglionated intestine is surrounded distally and proximally by aganglionosis [8, 9].

A second severe GI motility disorder, primarily affecting the small intestine is pediatric intestinal pseudo‐obstruction (PIPO), a serious GI dysfunction presenting with intestinal impediment without a definite mechanical blockage [4, 10, 11]. Subjects with PIPO might display diffuse alterations in one or more components of the neuromusculature of the GI tract [4]. PIPO has been reported as isolated cases or as an inherited disorder with a family history, although the dominantly inherited form of PIPO seems to be quite uncommon [12]. In summary, PIPO represents a relatively rare disorder with heterogeneous etiology, is difficult to treat, and causes significant morbidity [2, 11]. It has also been suggested that in addition to alterations of the neuronal and muscular compartments, GI motility disorder can be caused by an insufficient mesh of connective tissue located between the longitudinal and circular muscle layers of the muscularis propria, i.e., desmosis coli [13, 14, 15].

In most cases, the clinical manifestations of PIPO and HSCR start within the first months to the first year of life [1, 6, 10]. When the diagnosis is suspected, it might be confirmed by the use of intestinal transit analysis and manometric studies, and when possible, by histopathological analysis of suction biopsies and/or full‐thickness intestinal biopsies obtained during surgery [2, 16].

A significant component of the neuronal compartment in the GI tract in the ganglia are glial cells. Pathology in glia have been linked to alterations in a variety of proteins; among others is SOX10, SRY‐Box transcription factor 10, and various missense mutations of the *SOX10* gene have been reported to date [17, 18, 19]. SOX10 belongs to a family of 20 “Sex‐determining region Y‐related high‐mobility group box‐containing” SOX‐proteins [20]. The SOX10 transcription factor is a characteristic marker for neural crest derivatives, including sensory, autonomous, enteric ganglia and Schwann cells, among others [21]. *SOX10* mutations have been reported to be involved in Waardenburg syndrome (WS), which is caused by abnormal neural crest migration and further classified into four subtypes, WS types 1 to 4 [20, 22]. The incidence of WS is around 1/212,000, with autosomal dominant or recessive inheritance and 20% penetrance [22]. One of the subtypes, WS4, displays symptoms of PIPO and/or HSCR. In the WS4 group, there is a more severe subtype, PCWH syndrome, i.e., Peripheral demyelinating neuropathy, Central dysmyelinating leukodystrophy, WS and HSCR disease [20]. Clinically, PCWH syndrome is characterized by alterations in the peripheral and central nervous system, pigmentation abnormalities, hearing loss and HSCR [19].

The immunohistochemical (IHC) marker for SOX10 protein has been widely used in tumor diagnostics for about 20 years [22, 23, 24]. The SOX10 protein was first reported to be expressed by glial cells in 1998 [25]. Since then antibodies directed to SOX10 have been used when assessing glial cells in samples from the GI tract in human subjects with PIPO and/or HSCR [26, 27, 28, 29].

We had the unique opportunity to assess histopathological and immunohistochemical alterations in the GI tract of two children with WS4. Both children presented with classical clinical signs of WS with white forelock, PIPO, and hearing loss.

## 2 Materials and Methods

Two subjects, both with a clinical diagnosis of WS4, one with PCWH syndrome and one with Waardenburg‐Shah syndrome (WSS), underwent surgery at the local hospital. The clinical, pathological or genetic observations in these subjects have not been reported previously. In addition, four “age‐matched” subjects without suspicion of WS or any neuromuscular GI disorder were identified from the local Laboratory Registry and were used for comparison. Demographics of these six subjects are summarized in Table 1. The cases were received by the laboratory over a time span of several years. Permission for this study was obtained from the National Ethical Committee (2024/02830–01), and in the cases with genetic alterations informed consent were obtained from the parents. In all six cases, samples for diagnostic purposes had been obtained closely after birth during surgery from various locations in the GI tract (Table 1).

**TABLE 1:** Demographics of the included subjects.

Subject	Age at operation	Gender	Reason for surgery	Site of biopsy small intestine	Site of biopsy large intestine
C1	2 days	Female	Colon atresia	Distal ileum	
C2	20 days	Male	Ileus	Distal ileum	
C3	19 days	Male	Ileus		Transversum
C4	1 day	Male	Diaphragmatic hernia*		Transversum
PCWH	22, 30 days	Female	1. Malrotation 2. Ileus	Jejunum Proximal ileum Distal ileum	Ascendens descendens Sigmoideum rectum
WSS	3 days	Male	Neonatal ileus	25 cm from Lig. Treitz 75 cm from Lig. Treitz 95 cm from Lig. Treitz	Caecum

Abbreviations: C, control; Lig, ligamentum; PCWH, peripheral demyelinating neuropathy‐central dysmyelinating Leukodystrophy‐Waardenburg syndrome‐Hirschsprung disease; WSS, Waardenburg‐Shah syndrome.

* Resection of colon due to thin and flaccid intestinal wall.

The samples were received by the laboratory fresh on gauze saturated with saline solution. Most of the assessed full‐thickness samples were small, measuring up to 5 mm in diameter. After gross examination, the samples were placed in buffered formalin for fixation and subsequently embedded in paraffin. Routine diagnostics was carried out using histological sections of 4 μm thickness for histological stains. While assessing the Hematoxylin and Eosin (HE) stained sections the length of MP was measured using a microscope mounted camera (Olympus BX45, SC50) in the section deemed most representative. The length was defined as the length of the intersection between the inner and the outer muscle layers of the muscularis propria (Table 2). The most representative section was also selected for additional stains (Van Gieson and elastin, Periodic Acid‐Schiff (PAS), PAS‐diastase, Giemsa). For the Congo red stain 15 μm thick sections were used. In addition, 3 μm thick sections were cut for IHC stains as given in Table 3. All stains were carried out using automated stainers. Some IHC stains (Hu C/D, SOX10, CD117) were repeated for this study so that the outcome would be comparable.

**TABLE 2:** Description of the samples assessed and the number of ganglion and glial cells in the specimens.

Subject	Site of biopsy	Number of sections	Orientation	Length of MP in mm/section*	Number of ganglion cells/section**	Number of ganglion cell/length of MP	Number of glial cells/section***	Number of glial cell/length of MP
C1	Ileum NOS	7	Transverse	29.5	831	28.2	2586	87.7
C2	Distal ileum	9	Transverse	24.7	696	28.2	1038	42.0
C3	Transversum	16	Transverse	2.7	139	51.5	274	101.5
C4	Transversum	20	Transverse	26.9	931	34.6	2049	76.2
PCWH	Jejunum	6	Transverse	6.3	60	9.5	184	29.2
	Proximal ileum	18	Transverse	1.8	16	8.9	25	13.9
	Distal ileum	27	Transverse	14.4	14	1.0	66	4.6
	Ascendens	8	Transverse	5.3	12	2.3	122	23.0
	Descendens	11	Unclear	4.3	19	4.4	87	20.2
	Sigmoideum	8	Unclear	2.9	12	4.1	69	23.8
	Rectum	44	Unclear	1.7	20	11.8	30	17.6
WSS	25 cm from Lig. Treitz	11	Unclear	12.8	0	0	31	2.4
	75 cm from Lig. Treitz	10	Unclear	17.1	43	2.5	85	5.0
	95 cm from Lig. Treitz	10	Unclear	5.5	0	0	19	3.5
	Caecum	10	Unclear	3.4	0	0	46	13.5

Abbreviations: C, control; Lig, ligamentum; MP, myenteric plexus; PCWH, peripheral demyelinating neuropathy‐central dysmyelinating Leukodystrophy‐Waardenburg syndrome‐Hirschsprung disease; WSS, Waardenburg‐Shah syndrome.

* In HE stained section.

** In HuC/D stained section.

*** In SOX10 stained section.

**TABLE 3:** Immunohistochemistry.

Antibodies	Clone	Manufacturer	Dilution	Pretreatment	Stainer
B‐lymphocyte (CD20)	L‐26	Dako–Agilent	RTU	pH 8.5^1^	DAKO/Omnis
Calretinin	566	Novocastra	1:100	pH 8.5^1^	DAKO/Omnis
Desmin	D33	Dako–Agilent	RTU	pH 8.5^1^	DAKO/Omnis
ELAV–like protein 4 (HuC/D)	16A11	Invitrogen	1:1500	pH 8.5^1^	DAKO/Omnis
Leucocyte common antigen (LCA)	2B11 + PD7/26	Dako–Agilent	RTU	pH 8.5^1^	DAKO/Omnis
Macrophage, (CD68)	KP1	Dako–Agilent	RTU	pH 8.5^1^	DAKO/Omnis
Peripherin	PJM50	Novocastra	1:100	pH 8.5^1^	DAKO/Omnis
Smooth muscle actin (SMA)	1A4	Dako–Agilent	1:400	pH 8.5^1^	DAKO/Omnis
SRY‐Box transkription factor 10 (SOX10)	EP 268	Cell Marque	RTU	pH 8.5‐9.0^2^	Ventana BenchMark Ultra
T‐lymphocytes (CD3)	Polyclonal	Dako–Agilent	RTU	pH 6.0^3^	DAKO/Omnis
Tyrosine–protein kinase KIT (CD117)	Polyclonal	Dako–Agilent	RTU	pH 8.5^1^	DAKO/Omnis

*Note:* Buffers ^1^Tris/EDTA, ^2^Cell conditioning solution 1, ^3^Citrate buffer.

Abbreviations: DAKO/Omnis‐EnVision FLEX detection system; RTU, ready to use; Ventana BenchMark Ultra–MU ultraView Universal DAB Detection Kit.

The selection of commercial antibodies regarding these three proteins was based on commercial availability, performance and reproducibility. The antibody directed to SOX10 and c‐kit/CD117 are frequently used in our and other laboratories [23, 24, 30]. Furthermore, the antibody directed to SOX10 has been used for 20 years to visualize glial cells in the gut [25, 26, 27]. C‐kit/CD117 was used to visualize the interstitial cells of Cajal (ICC). For ganglion cells, a pan‐neuronal marker HuC/D, routinely used by us and others, was chosen [31, 32]. All stained sections were assessed by light microscope magnification ranging from ×40 to ×400. The counts of “HuC/D‐labeled” and “SOX10‐labeled” cells in the MP region was carried out manually.

## 3 Results

**FIGURE 1:**
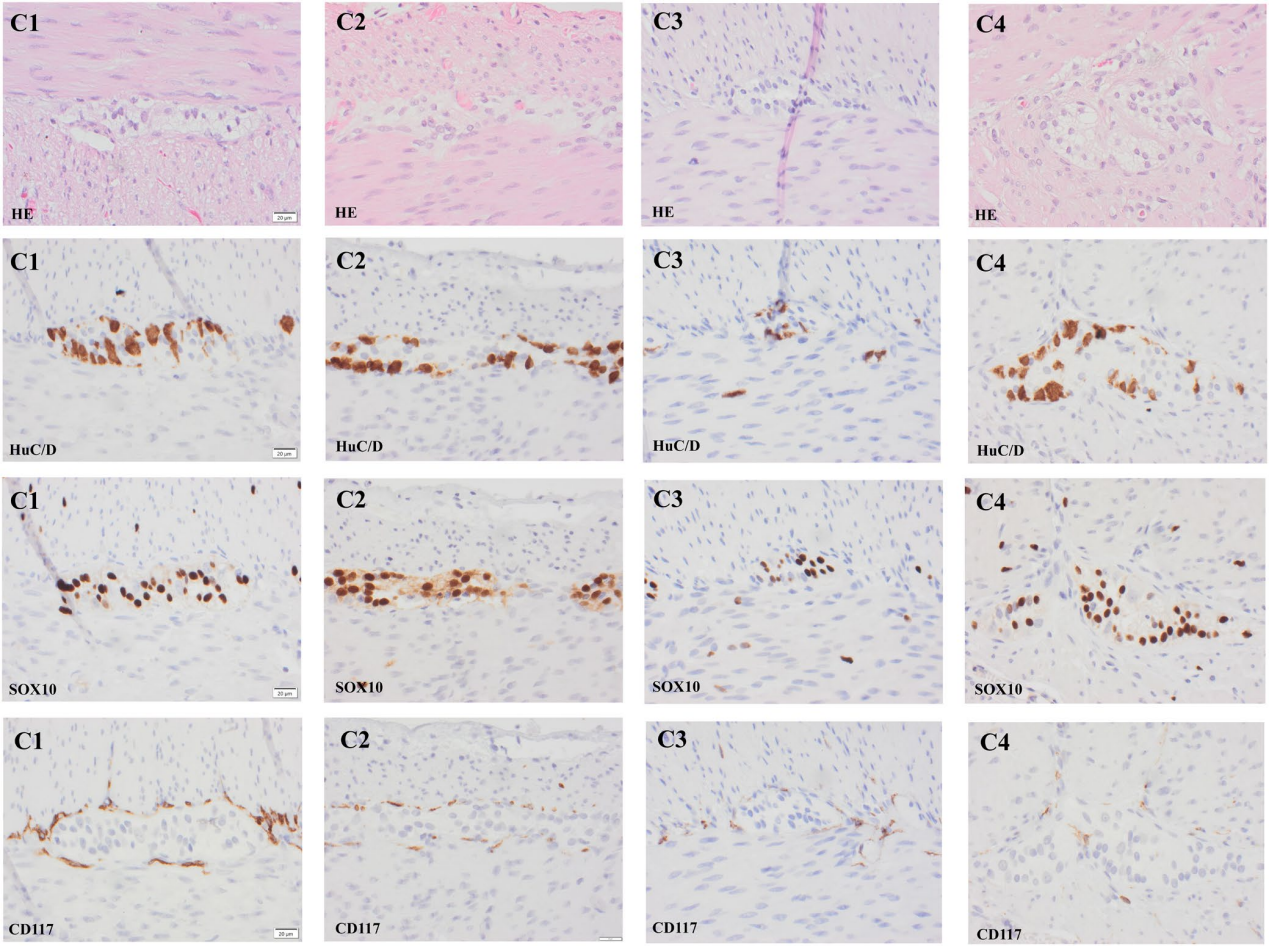
Photomicrographs of samples obtained from ileum (C1, C2) and colon (C3, C4) from four subjects lacking indication of Waardenburg Syndrome (Tables 1, 2). The ganglion is seen in hematoxylin–eosin (HE) stain centrally, between the circular layer of muscularis propria in the upper part of the photomicrograph and the longitudinal muscle layer. Note the perinuclear cytoplasmic labeling of ganglion cells with the pan neuronal marker, RNA binding protein HuC/D, the nuclear labeling in glial cells with the SRY Box transcription factor 10 (SOX10) and the cytoplasmic labeling of Cajal cells with proto‐oncogene c‐KIT encoding the receptor tyrosine kinase KIT also known as CD117. Note numerous HuC/D labeled ganglion cells, glial cells and cells of Cajal in the samples.

Four control subjects were available for this study. In total 2 samples from the small and 2 samples from the large intestine were investigated. In Figure 1 C1‐2 (ileum) and C3‐4 (colon) numerous “HuC/D‐labeled” ganglion cells, “SOX10‐labeled” glial cells and “CD117‐labeled” Cajal cells are visualized. In Table 2, the number of sections assessed, length of the MP, number of “HuC/D‐labeled” ganglion and “SOX10‐labeled” glial cells are given. The number of these cell in relation to the length of MP was calculated and given as the number of cells per unit length. In the control cases, the number of ganglion cells ranged from 28.2 to 51.5 cells/mm of MP and the number of glial cells ranged from 42.0 to 101.5 cells/mm of MP.

Both children with WS were delivered at roughly the same gestational age (GA): the female subject with PCWH at 38 + 2 weeks and the male subject with WSS at 38 + 3 weeks. Both presented directly after birth symptoms of neonatal ileus.

**FIGURE 2:**
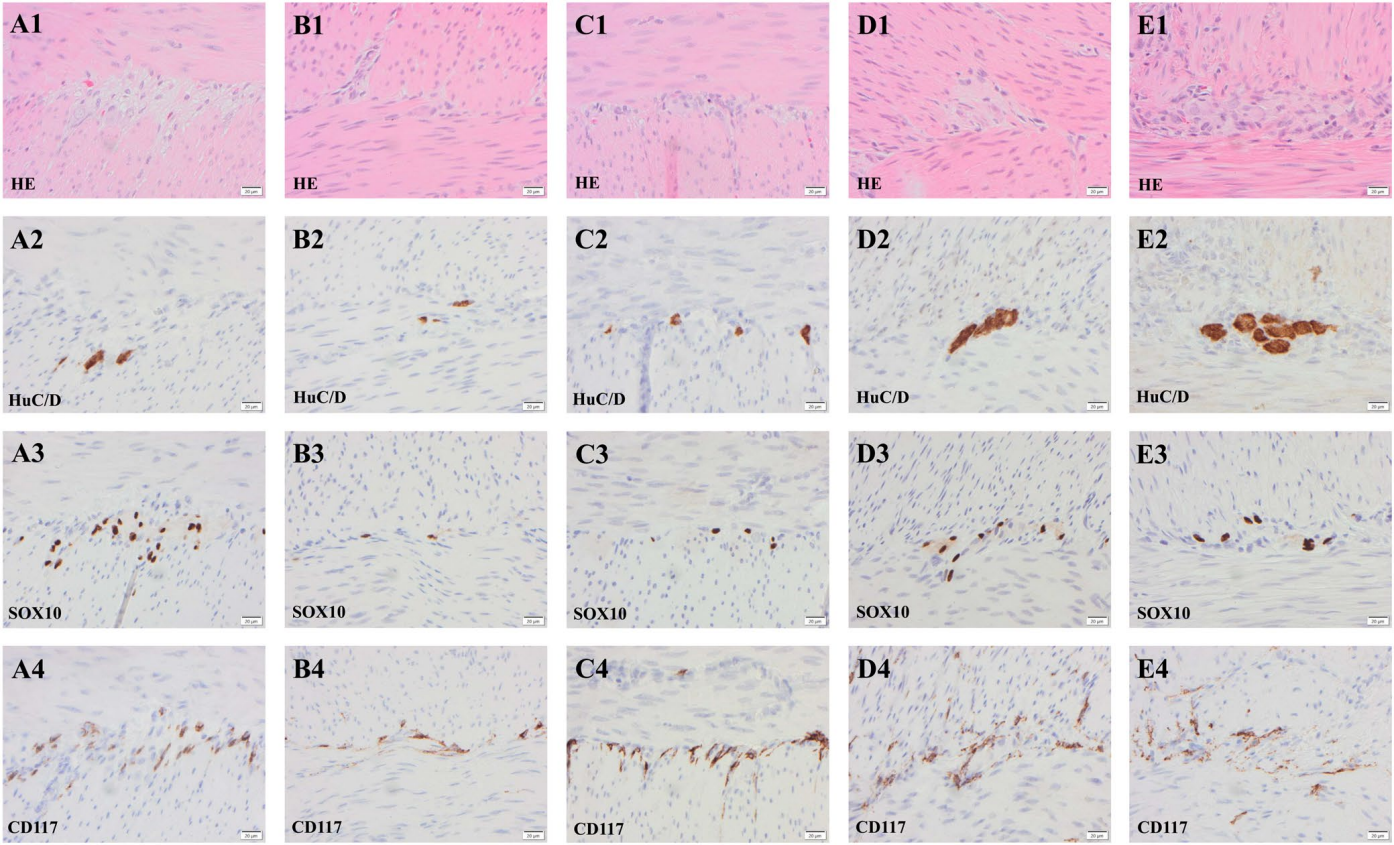
Photomicrographs of samples obtained from the gastrointestinal tract of the subject with Peripheral demyelinating neuropathy‐central dysmyelinating leukodystrophy‐Waardenburg syndrome‐Hirschsprung disease (PCWH). The sample obtained from jejunum (A1–A4), from proximal ileum (B1–B4), from distal ileum (C1–C4), from sigmoideum (D1–D4 and from rectum (E1–E4). The ganglion is seen in hematoxylin–eosin (HE) stain centrally, between the circular layer of muscularis propria in the upper part of the photomicrograph and the longitudinal muscle layer. Note the perinuclear cytoplasmic labeling of ganglion cells with the pan neuronal marker, RNA binding protein HuC/D, the nuclear labeling in glial cells with the SRY Box transcription factor 10 (SOX10) and the cytoplasmic labeling of Cajal cells with proto‐oncogene c‐KIT encoding the receptor tyrosine kinase KIT also known as CD117. Note the moderate decrease in the number of both HuC/D labeled neurons and “SOX10‐labeled” glial cells in the PCWH case when compared to the control (Figure 3). Bar 20 μm.

The investigation of the female subject with PCWH, revealed that she suffered from malrotation of the gut, and she underwent surgery at the age of 22 days. After the operation, her gut still did not function normally; hence, she had another operation at the age of 30 days during which an ileostomy was created. Samples were taken during both the first (rectum) and the subsequent operation (small and large intestine). Full‐thickness intestinal biopsies were obtained from the following locations: jejunum 37 cm from the duodenum (Figure 2 A1‐4), proximal ileum (Figure 2 B1‐4), distal ileum 16 cm from the ileocecal valve (Figure 2 C1‐4), colon ascendens and descendens (not visualized), and sigmoideum (Figure 2 D1‐4). From the rectum, a suction biopsy was taken including muscularis propria (Figure 2 E1‐4). Ganglia within MP were readily seen in “HE‐stained” section. Ganglion cells labeled with HuC/D antibody were observed in all assessed samples ranging from 1.0 to 11.8 cells/mm of MP (Table 2, Figures 1 and 2). Aganglionotic segments were not observed in this case. No hypertrophic nerves were seen in the sample from rectum or in any other location. “SOX10‐labeled” glial cells were seen in all locations, but the number was lower when compared to the control cases ranging from 4.6 to 29.2 cells/mm of MP (Table 2, Figures 1 and 2). The number of “CD117‐labeled” ICC was comparable with what was seen in the controls (Figures 1 and 2). A genetic analysis was carried out which revealed a *SOX10* mutation (c.769A>T, p.Lys257*) consistent with the diagnosis of WS4 subtype PCWH.

**FIGURE 3:**
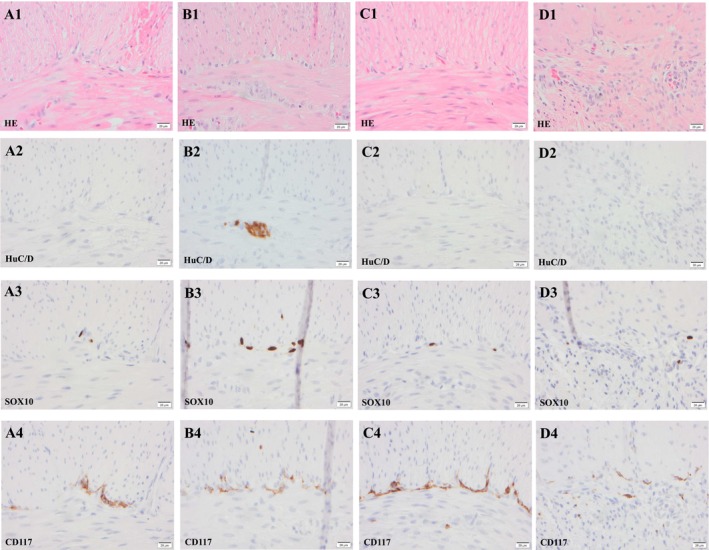
Photomicrographs of samples obtained from gastrointestinal tract from the subject with Waardenburg‐Shah syndrome. The samples were obtained from the small intestine 25 cm from ligamentum Treitz (A1–A4), 75 cm from ligamentum Treitz (B1–B4) and 95 cm from ligamentum Treitz (C1–C4) and from caecum (D1–D4). The ganglion is seen in hematoxylin–eosin (HE) stain centrally, between the circular layer of muscularis propria in the upper part of the photomicrograph and the longitudinal muscle layer. Note the perinuclear cytoplasmic labeling of ganglion cells with the pan neuronal marker, RNA binding protein HuC/D, the nuclear labeling in glial cells with the SRY Box transcription factor 10 (SOX10) and the cytoplasmic labeling of Cajal cells with proto‐oncogene c‐KIT encoding the receptor tyrosine kinase KIT also known as CD117. Note complete absence of HuC/D labeled ganglion cells in three of the four locations as well as a severe reduction of “SOX10‐labeled” cells. Bar 20 μm.

The male subject with WSS developed a distended proximal small intestine and underwent surgery at the age of 3 days, during which a stoma was created in the upper part of the small intestine. Full‐thickness biopsies were obtained from the small intestine at 25 cm (Table 2, Figure 3 A1‐4), 75 cm (Figure 3 B1‐4), and 95 cm (Figure 3 C1‐4) from the ligamentum Treitz as well as from the caecum (Figure 2 D1‐4). The ganglia within MP were difficult to identify in “HE‐stained” sections. Ganglion cells were seen (HuC/D) only in the sample obtained from the small intestine 75 cm from ligamentum Treitz at the level of 2.5 cells/mm of MP (Table 2, Figure 3). Contrary to the above, in other locations of the small intestine and caecum, HuC/D labeled ganglion cells were missing, aganglionosis. The number of SOX10 labeled glial cells was sparse ranging from 2.4 to 13.5 cells/mm MP (Table 2, Figure 3). The number of CD117 labeled ICC was comparable with what was seen in the controls (Figures 1 and 3). A genetic analysis was carried out which revealed a homozygotic endothelin 3 mutation (*EDN3*) (c.472C>T, p.Arg158Cys). To our knowledge, this particular *EDN3* mutation has not previously been associated with WS4, WSS, or PCWH syndrome.

In summary, in the child with PCWH syndrome, hypoganglionosis with HuC/D labeling was observed in both the small and large intestines excluding the rectum. This was accompanied by a parallel reduction in the number of “SOX10‐labeled” glial cells. In contrast, the number of ICCs labeled with CD117 seemed unaffected. For the child with WSS, with HuC/D labeling a general aganglionosis with a “skipped segment” in the proximal ileum was observed in the intestine, accompanied by a parallel reduction in the number of “SOX10‐ labeled” glial cells. The number of ICCs labeled with CD117 seemed unaffected.

Based on the assessment of all slides stained with various histological and IHC stains, the visceral compartment, including the mucosa, submucosa, muscularis, and subserosa, appeared unremarkable in both the study and control cases. In line with what was seen within the region of MP the number of “IHC‐labeled” ganglion and glial cells was reduced in the submucosa in the subject with PCWH. In the subject with WSS the ganglion cells were missing in submucosa in the regions of aganglionosis within MP and there were few glial cells. In the skipped segment in the WSS case few ganglion and glial cells were observed in submucosa. No hypertrophic nerves were seen in any of the cases, in all subjects a meshwork of connective tissue was seen between the circular and longitudinal muscle layers and no signs of ganglionitis were observed.

Besides a non‐functioning gut, the clinicians noted pigmentation alterations and a white forelock in both diseased cases and they both had hearing deficiency consistent with what is expected for WS4 [33].

## 4 Discussion

Here, we report the histological findings observed in two newborn children: one with clinically defined PCWH syndrome (*SOX10*; c.769A>T, p.Lys257*) and one with clinically defined WSS (*EDN3*; c.472C<T, p.Arg158Cys). Both *SOX10* and *EDN3* mutations have been associated with WS [19, 22, 34].

We focused our assessment on ganglion cells, glial cells and ICCs in the MP using specific markers, HuC/D, SOX10 and CD117, and we compared our findings with four “age‐matched” controls [35]. The length of MP varied from 1.8 to 29.5 mm. We counted the number of ganglion cells and glial cells and calculated the number of these cells per unit length of MP (Table 2). The value of ganglion and glial cells per unit of length was considered being descriptive and reproducible when assessing the number of these cells. We are confident that in our subjects with PCWH and WSS, there is a notable reduction in glial cells and ganglion cells observed already at birth.

In experimental animal studies, it has been shown that the migration, proliferation, and differentiation of enteric neural crest cells (ENCC) requires glial cell‐derived neurotrophic factor (GDNF) signaling and EDN3 molecules [36, 37, 38, 39]. In the gut, the GDNF and EDN3 molecule, have been described to be secreted primarily by mesenchymal cells [38, 39]. During embryological development the vagal ENCC (vENCC) populates the gut by intramural rostrocaudal migration, this is followed by the “trans‐mesenteric” migration of vENCC from the small to the large intestine driven by long‐range chemoattraction [36]. This allows vENCC to bypass portions of the intestine during their rostrocaudal migration [36, 40]. This crossing of vENCC from the small to the large intestine via the mesentery creates a transient, physiological skipped segment during embryological development [36]. Failure to back fill the bypassed intestine has been proposed as an explanation for the skipped segments observed in some HSCR cases [40]. In addition to these vENCC, sacral derived ENCC (sENCC) have been described to colonize the distal hindgut [41].

In all sections from the patient with PCWH syndrome, ganglion cells were observed using the HuC/D stain, ruling out HSCR, i.e., aganglionosis. The rejection of the diagnosis of HSCR was further supported by the substantial number of “HuC/D‐labeled” ganglion cell (11.8 cells/mm MP) seen in the rectum and the lack of hypertophic nerves in this location. Noteworthy, in samples from other parts of the GI tract the number of ganglion cells was notably reduced, i.e., hypoganglionosis, compared to our control cases, as seen in Table 2. Congruent with our results, in 2018 Akutsu and colleagues described a PCWH case with a *SOX10* mutation (p.Ser282GlnfsTer12) exhibiting severe hypoganglionosis [42]. The *SOX10* mutations in these two PCWH cases are not the same. The unexpected observation of a substantial number of ganglion cells in rectum compared to the hypoganglionosis in other parts of the intestine seen in our PCWH case might be explained by the existence of the sENCC [41].

In contrast, in the patient with clinical WSS syndrome with an *EDN3* mutation, no HuC/D labeled ganglion cells were seen in the jejunum, distal ileum and caecum, consistent with segmental aganglionosis, i.e., HSCR. Similar outcomes, aganglionosis in gut samples, have been reported previously in cases with WSS [43, 44]. Noteworthy, in our case a few ganglion cells, 2.5 cells/mm MP, were observed in the proximal ileum, i.e., skipped segment, an alteration previously described in association with HSCR [8, 9, 40, 45].

The pathoethiological mechanisms leading to aganglionosis with a skipped segment seen in our WSS case mediated by mutations in the *EDN3* gene is complex. It is known that during early development *EDN3* plays an important role not only in the migration but also in the proliferation and differentiation of the ENCC [45, 46]. Since the skipped segment in our WSS case was located in the small intestine it cannot be explained by altered “trans‐mesenteric” migration as has been suggested for HSCR [40, 45].

In both our subjects, a severe reduction of glial cells labeled with SOX10 was observed in all samples compared to “age‐matched” controls. This outcome mediated by the *SOX10* and *EDN3* mutations might be related to low or sparse extent of GDNF and/or EDN3 molecule, thus the ENCC are not chemoattracted to their presumed final destinations and in addition to this their differentiation is altered and their survival hampered [38, 39].

In our control cases, the number of “CD117‐labeled” ICCs was relatively high in the ileum and moderate in the colon, which is consistent with what has been published previously [47]. In both our cases with WS4 syndrome the number of ICCs labeled with CD117 was in line with what was seen in the normal cases at this age.

There are relatively few published studies on humans that include histological assessment of gut specimens from subjects with various types of PIPO. Samples received by the pathology laboratory are in some cases taken from a GI tract location in relation to functional disturbance rather than in relation to a presumed disease entity. The age and gender of the subjects differs. The samples, usually small, are obtained either fixed or fresh on gauze and an optimal orientation is not always possible. To visualize cells and cell compartments various antibodies and IHC staining techniques are applied. Thus, when carrying out a PubMed search on the topic it is difficult to find studies with sampling and processing of the tissue comparable to our study. Overall, the results obtained are influenced by the mutations registered, the GI samples assessed and the methods used. As discussed above, results in line with ours have been reported; however, other studies have described WS4 subjects with GI motility disorder but with persistence of ganglion cells [48, 49, 50].

## 5 Pitfalls and Shortcomings

Our study is carried out on diagnostic samples that in general contain significant shortcomings. The stainings carried out are part of the routine diagnostics and are thus well controlled, reproducible and reliable. The shortage of age‐ and gender‐matched controls is a major weakness. Our controls were operated due to a GI alterations but lacked, based on our assessment, signs of neuroglial alterations. The location of gut samples obtained from our controls were not fully in line with the locations of gut samples obtained from our two WS4 cases. An additional shortcoming is that several of our samples were small and in some cases the orientation of the sample was not reliably assessed (Table 2).

## 6 Conclusion

Here, we show that the number of both glial cells and ganglion cell was reduced in our two subjects with WS4. The female subject with PCWH with a *SOX10* mutation (c.769A>T, p.Lys257*) displayed reduction in the number of glial cells and severe hypoganglionosis, however with ganglion and glial cells in the rectum, efficiently excluding the diagnosis HSCR. The male subject with WSS with an *EDN3* mutation (c.472C<T, p.Arg158Cys) displayed segmental aganglionosis, i.e., HSCR, with a skipped segment. The ICC seemed to be largely unaffected in our cases. In summary, we describe two newborn children with similar initial clinical presentation, neonatal ileus but with differing genetical alteration and histopathological findings.

## Author Contributions

Conception and design: Björn Ersson and Irina Alafuzoff. Medical record chart review: Elisabet Gustafson and Johan Danielson. Review of literature and data collection: Björn Ersson and Irina Alafuzoff. Drafting of the manuscript: Björn Ersson and Irina Alafuzoff. Revision of key components of the manuscript: All authors. Final approval of the manuscript: All authors.

## Ethics Statement

The study was approved by the national ethical committee # 2024–02830‐1, informed consent was obtained from the parents of both patients.

## Conflicts of Interest

The authors declare no conflicts of interest.

## Data Availability

Data sharing is not applicable to this article as no new data were created or analyzed in this study.
